# Thyroid-Related Protein Expression in the Human Thymus

**DOI:** 10.1155/2017/8159892

**Published:** 2017-03-02

**Authors:** Mi Jeong Kim, So Won Oh, Hyewon Youn, Juri Na, Keon Wook Kang, Do Joon Park, Young Joo Park, Ja June Jang, Kyu Eun Lee, Kyeong Cheon Jung, June-Key Chung

**Affiliations:** ^1^Department of Nuclear Medicine, Seoul National University College of Medicine, Seoul, Republic of Korea; ^2^Laboratory of Molecular Imaging and Therapy, Cancer Research Institute, Seoul National University College of Medicine, Seoul, Republic of Korea; ^3^Tumor Microenvironment Global Core Research Center, Seoul National University College of Medicine, Seoul, Republic of Korea; ^4^Department of Nuclear Medicine, Seoul National University Boramae Hospital, Seoul, Republic of Korea; ^5^Cancer Imaging Center, Seoul National University Cancer Hospital, Seoul, Republic of Korea; ^6^Department of Biomedical Sciences, Seoul National University College of Medicine, Seoul, Republic of Korea; ^7^Department of Internal Medicine, Seoul National University College of Medicine, Seoul, Republic of Korea; ^8^Department of Pathology, Seoul National University College of Medicine, Seoul, Republic of Korea; ^9^Department of Surgery, Seoul National University College of Medicine, Seoul, Republic of Korea

## Abstract

Radioiodine whole body scan (WBS), related to sodium iodide symporter (NIS) function, is widely used to detect recurrence/metastasis in postoperative patients with thyroid cancer. However, the normal thymic uptake of radioiodine has occasionally been observed in young patients. We evaluated the expression of thyroid-related genes and proteins in the human thymus. Thymic tissues were obtained from 22 patients with thyroid cancer patients of all ages. The expression of NIS, thyroid-stimulating hormone receptor (TSHR), thyroperoxidase (TPO), and thyroglobulin (Tg) was investigated using immunohistochemistry and quantitative RT-PCR. NIS and TSHR were expressed in 18 (81.8%) and 19 samples (86.4%), respectively, whereas TPO was expressed in five samples (22.7%). Three thyroid-related proteins were localized to Hassall's corpuscles and thymocytes. In contrast, Tg was detected in a single patient (4.5%) localized to vascular endothelial cells. The expression of thyroid-related proteins was not increased in young thymic tissues compared to that in old thymic tissues. In conclusion, the expression of NIS and TSHR was detected in the majority of normal thymus samples, whereas that of TPO was detected less frequently, and that of Tg was detected rarely. The increased thymic uptake of radioiodine in young patients is not due to the increased expression of NIS.

## 1. Introduction

Thyroid hormone is synthesized by a pathway involving several proteins. Iodine is transported into the thyroid follicular cells through the sodium iodide symporter (NIS), where the thyroid hormone is produced by thyroid-related proteins including thyroid-stimulating hormone receptor (TSHR), thyroperoxidase (TPO), and thyroglobulin (Tg) [1]. Because the expression of these proteins is somewhat persistent in differentiated thyroid cancer (DTC), radioiodine whole body scan (WBS) has been used to detect recurrence and metastasis after thyroidectomy in patients with DTC [2, 3].

Radioiodine uptake in the thymus was observed in one-fourth of young patients without any evidence of metastasis or recurrence in the thymus, which can be misinterpreted as metastatic/recurrent thyroid cancer [4, 5]. These reports imply the presence of NIS in the normal thymus. To the best of our knowledge, a single study has been published describing the expression of thyroid-related genes, such as NIS, TPO, TSHR, and Tg in the thymus of only two cases [6]. However, they did not study the differential expression of thyroid-related genes and proteins according to age.

In the present study, the expression of the thyroid-related proteins and genes was evaluated in the normal thymic tissues obtained from patients undergoing surgery for papillary thyroid carcinoma (PTC). The expression of thyroid-related genes and proteins was also evaluated in thymic tissues obtained from patients of different ages to determine whether the expression of thyroid-related genes is increased in the young thymus.

## 2. Materials and Methods

### 2.1. Patients and Samples

Twenty-two female patients with PTC were enrolled and classified into four subgroups according to age: 20–29 years (20s, *n* = 5), 30–39 years (30s, *n* = 6), 40–49 years (40s, *n* = 5), and over 50 years (over-50s, *n* = 6). The thymic tissues were in close proximity to the inferior thyroid pole and were obtained during central neck dissection adjunctive to total thyroidectomy. A pathologist confirmed the histology of normal thymic tissues without cancer metastasis using hematoxylin and eosin (H&E) staining. Among 22 patients, mRNA expression levels were examined in 13 patients in whom the amount of isolated RNA was sufficient for quantitative real-time polymerase chain reaction (qPCR) analysis. These included three patients in the 20s, five in the 30s, three in the 40s, and two in the over-50s group. Normal thyroid tissues and thyroid tissue obtained from a patient with Graves' disease were used as positive controls.

Written informed consent was obtained from each patient, and the Institutional Review Board at the Seoul National University Hospital approved this study.

### 2.2. Immunohistochemistry

Paraffin-embedded tissues were cut in 4 *μ*m thick sections, treated with xylene, and, subsequently, rehydrated. The tissue sections were heated using a microwave in 10 mM sodium citrate buffer for 8 min for antigen retrieval and then blocked with normal goat or horse serum (Vector Laboratories Ltd., Burlingame, CA). The tissue sections were incubated with primary antibodies for NIS (goat polyclonal antibody (sc-48052); Santa Cruz Biotechnology, CA), Tg (rabbit polyclonal antibody (A0251); Dako, Glostrup, Denmark), TSHR (goat polyclonal antibody (sc-7816); Santa Cruz Biotechnology), TPO (rabbit polyclonal antibody (NBP1-80670); Novus Biologicals, Littleton, CO), Pan-Cytokeratin (mouse monoclonal antibody (sc-53403); Santa Cruz Biotechnology), and CD31 (mouse monoclonal antibody (ab9498); Abcam, Cambridge, UK) overnight at 4°C. We used goat normal serum (Vector Laboratories Ltd.) and isotype mouse and rabbit control IgG (Life Technologies, Inc.) as a negative control instead of primary antibodies. After washing, the tissue sections were incubated with biotinylated secondary anti-rabbit, anti-goat, or anti-mouse antibodies for 1 h at room temperature and then treated with the Elite ABC kit reagent (Vector Laboratories Ltd.) for 30 min. For color development, the tissue sections were stained with the 3,3′-diaminobenzidine peroxidase substrate solution (Vector Laboratories Ltd.) and counterstained with Mayer's hematoxylin (Sigma-Aldrich). For negative controls, the tissue sections were incubated with isotype control IgG instead of primary antibodies. The tissue sections were examined under a light microscope after mounting with Permount mounting medium.

Immunoreactivity was classified according to IHC staining intensity using a 4-point scoring system: 0, 1, 2, or 3 (for none, weak, moderate, or strong staining, resp.). Samples with scores of over 2 were considered as positively stained. IHC evaluations were performed blindly by two readers and conformed by a pathologist with an interobserver concordance of nearly 100%. The distribution rate of the stained area was assessed by counting the positively stained and total Hassall's corpuscles in the thymic tissues and expressed as the percentage of positive Hassall's corpuscles. The percentages of the positive samples in thymocytes were determined.

### 2.3. Quantitative Real-Time RT-PCR

Total RNA was extracted from thyroid and thymus tissues using TRIzol (Life Technologies, Inc.), according to the manufacturer's instruction. The RNA extracts were reverse transcribed to cDNA templates using random primers and Moloney murine leukemia virus (M-MLV) reverse transcriptase (Invitrogen, Carlsbad, CA), in a final volume of 20 mL at 42°C for 50 min followed by heating at 70°C for 15 min. qPCR was performed using the ABI 7500 Real-Time PCR System (Applied Biosystems, Foster City, CA). A 25 mL reaction mixture consisted of 5 mL cDNA template, 12.5 mL TaqMan Universal PCR master mix (Applied Biosystems, Foster City, CA), and 1.25 mL NIS or Tg primer probe mixture (Applied Biosystems, Foster City, CA). The following thermal cycler parameters were used for amplification: incubation at 50°C for 2 min, denaturation at 95°C for 10 min, 40 cycles of denaturation at 95°C for 15 s, and annealing/extension at 60°C for 1 min. The ΔΔCt method was used to calculate the mRNA levels. For endogenous control, RNA of human GAPDH was used, and all reactions were performed in triplicate. The mRNA expression levels of both NIS and Tg were examined in normal thyroid tissues, and the relative amounts of mRNA in thymic samples were expressed as percentages of levels found in normal thyroid tissues. For a negative control, distilled water was used instead of tissue-extracted cDNA templates.

### 2.4. Statistical Analysis

The intensity of IHC staining was evaluated using a scoring system and calculated as the percentages of each age group and all participants. The differences among age groups were compared using logistic regression analysis. The odds ratios and corresponding 95% confidence intervals (CI) were calculated as a measure of the difference according to the age group. A *p* value of <0.05 was considered statistically significant.

## 3. Results

### 3.1. H&E Staining of Human Thymic Tissues

**Figure 1:**
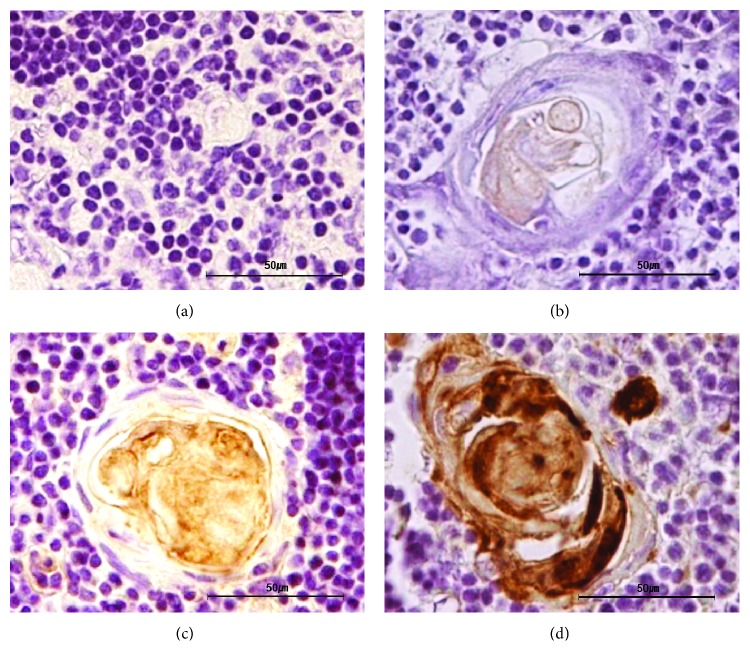
Expression of thyroid-related proteins in the thymus by immunohistochemistry with a 4-point scoring system. Representative images of score 0 (a), 1 (b), 2 (c), or 3 (d) are displayed. All images are magnified 400x, and the scale bars represent 50 *μ*m.

Histological analyses demonstrated that neither ectopic thyroid tissues nor metastatic thyroid cancer was found in the thymic tissues. The thymic tissues were involuted in older patients as the parenchyma was replaced by adipocytes, whereas the parenchyma remained in the thymic tissues of young patients. Representative 24-year-old, 39-year-old, 46-year-old, and 55-year-old thymic tissues stained with H&E are shown in Figure 1A, B, C, and D in Supplementary Material available online at https://doi.org/10.1155/2017/8159892, respectively. Distinguishing the thymic structures, including the capsule, thymic lobule, cortex, and medulla, was possible in 20- to 30-year-old thymic tissues (Supplemental Figure 1A and B). However, the thymus was involuted in most thymic tissues of patients more than 40 years old because the thymic compartments were reduced in size and adipocytes were present (Supplemental Figure 1C and D).

### 3.2. Incidence and Pattern of Thyroid-Related Protein Expression

**Figure 2:**
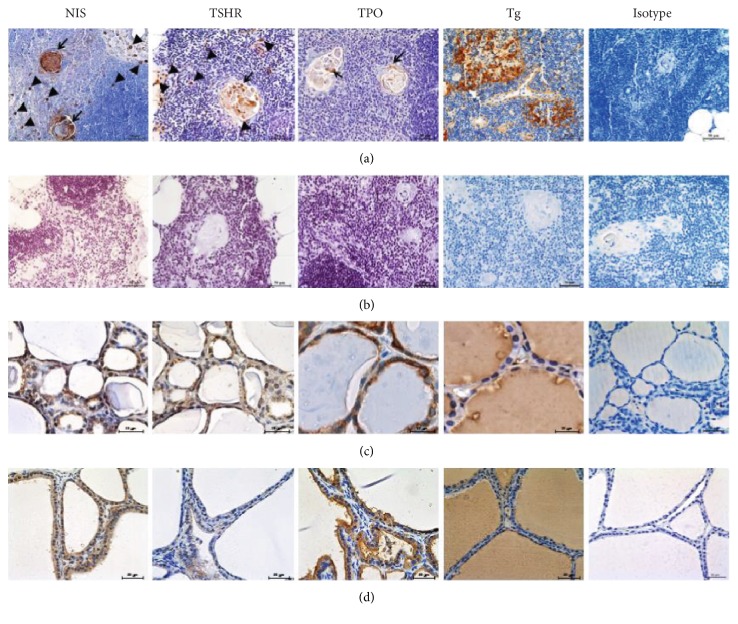
Expression of thyroid-related proteins in the thymus. Expression of thyroid-related proteins evaluated by immunohistochemistry. Representative images of thymic tissues that stained positive (a) or negative (b) for thyroid-related protein expression. The positive stained cells in Hassall's corpuscles (arrows) and in thymocytes (arrowheads) are indicated. The expression of thyroid-related protein was observed in normal thyroid tissue (c) and thyroid tissue obtained from a patient with Graves' disease (d) as a positive controls. Representative images of isotype control are shown as a negative control. All images are magnified 400x, and the scale bars represent 50 *μ*m (a, b, and d) and 20 *μ*m (c). NIS, sodium iodide symporter; TSHR, thyroid-stimulating hormone receptor; TPO, thyroid peroxidase; Tg, thyroglobulin.

Thyroid-related proteins were detected in the human thymus using IHC. Representative images of IHC staining intensity using a 4-point scoring system (0, 1, 2, or 3 for none, weak, moderate, or strong staining, resp.) are shown in Figure 1. The expression of NIS and TSHR was mainly observed in Hassall's corpuscles and in thymocytes of the thymic medulla (Figure 2(a)). TPO was only expressed in Hassall's corpuscles. Representative images of the thymic tissues staining positive and negative for thyroid-related protein expression are shown in Figure (2(a)) and (b), respectively. Representative 32-year-old thymic tissue positive for NIS and TPO, 24-year-old thymic tissue positive for TSHR, and 29-year-old thymic tissue positive for Tg are shown in Figure 2(a). Cells showing positive staining in Hassall's corpuscles (arrows) and in thymocytes (arrowheads) are indicated. Representative images of tissues staining positive for NIS and TSHR in both Hassall's corpuscles and thymocytes are shown. However, some samples showed positive staining only in Hassall's corpuscles. Thyroid-related protein expression was also observed in the normal thyroid tissue and in the thyroid tissue obtained from a patient with Graves' disease as positive controls (Figure 2(c)) and (d)). Representative images of isotype control in each sample are shown in Figures 2(a)–(d).

We confirmed NIS expression in human thymic tissues by Western Blot. The expression of nonglycosylated NIS (approximately 50 kDa) in human thymic lysates was mainly detected, and the expression of glycosylated NIS (87–110 kDa) in some thymus samples (patient numbers 55, 38, 48, and 42) was detected (Supplemental Figure 2). The expression of both glycosylated and nonglycosylated NIS in TPC-1 cells (Supplemental Figure 2) and human normal thymus obtained from patients with thyroid cancer (data not shown) was detected.

NIS and TSHR were expressed in 81.8% (18/22) and 86.4% (19/22) of the samples, respectively (Table 1). The average intensities of NIS and TSHR were 1.9 and 1.9, respectively. All samples positive for NIS and TSHR expressed these proteins in Hassall's corpuscles, except for NIS expression in one sample from a patient in 20s. NIS and TSHR were expressed in the thymocytes of some thymic tissues. To assess the stained areas, the percentages of positive Hassall's corpuscles among the total number of Hassall's corpuscles were calculated. The percentages of samples containing NIS-positive Hassall's corpuscles and TSHR-positive Hassall's corpuscles were 71.2% and 79.7%, respectively (Supplemental Table 1). NIS and TSHR were also expressed in 22.7% (5/22) and 68.2% (15/22) of thymocytes in the thymic samples, respectively (Supplemental Table 2).

TPO expression was less frequently observed (5/22, 22.7%), and the average intensity of TPO was 0.5 (Table 1). The percentage of samples containing TPO-positive Hassall's corpuscles was 14.1% of the samples (Supplemental Table 1), and no TPO-positive thymocytes were found in any samples (Supplemental Table 2).

**Figure 3:**
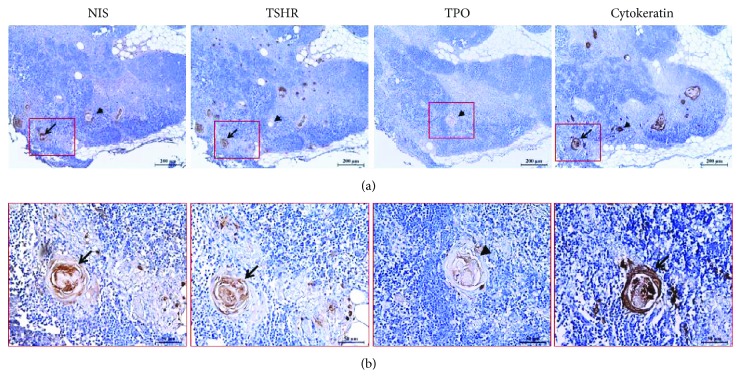
Localization of NIS, TSHR, and TPO in the thymus. The expression of NIS, TSHR, and TPO is localized to the Hassall's corpuscles (arrows and arrowheads) that are positively stained with cytokeratin. The low (a) and high (b) magnifications of the images are 100x and 400x, respectively. The scale bars represent 200 *μ*m (a) and 50 *μ*m (b). NIS, sodium iodide symporter; TSHR, thyroid-stimulating hormone receptor; TPO, thyroid peroxidase.

Additional staining with cytokeratin confirmed that NIS, TSHR, and TPO were distinctively localized to the Hassall's corpuscles (Figure 3). Hassall's corpuscles have been known to be stained with specific cytokeratin antibodies [7]. In Figure 3, arrows and arrowheads indicate the expression of NIS, TSHR, and TPO in a single Hassall's corpuscle, as seen in a series of thymic tissue sections obtained from a single patient. These results show that NIS, TSHR, and TPO are expressed in the same Hassall's corpuscle (arrowhead), although the expression frequencies of NIS, TSHR, and TPO are different in the same thymic tissue. As shown in Figure 3 and in Supplemental Table 1, the expression frequency of TPO was lower than that of NIS and TSHR in thymic tissues.

**Figure 4:**
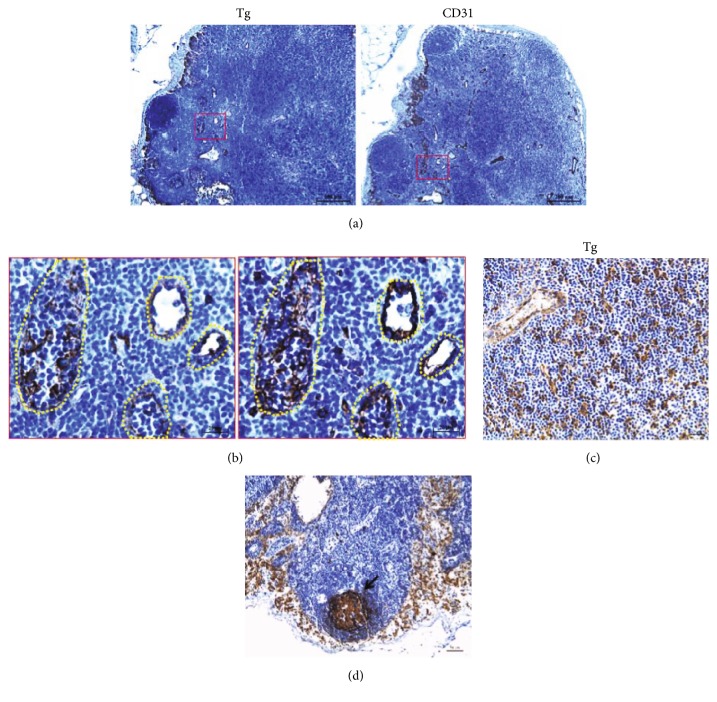
Expression of Tg protein in the Tg-positive thymus specimen. Thyroglobulin (Tg) was identified in the vasculature stained with the CD31 antibody (a and b). Tg was observed in thymocytes (c) and germinal center (d). The positive stained cells in the germinal center (arrows) are indicated. The low (a) and high (b) magnifications of the images are 100x and 400x, respectively, and (c) and (d) of the images are 400x. The scale bars represent 200 *μ*m (a), 20 *μ*m (b), and 50 *μ*m (c and d).

Unlike the other thyroid-related proteins, the expression of Tg was observed in the luminal structures that were lined with thin walls (Figure (2(a)). Furthermore, IHC data showed that Tg colocalized with CD31, indicating that Tg expression is localized to vascular endothelial cells (Figure 4(a)) and 4(b)). Some Tg-positive signals did not perfectly match with CD31-positive cells, suggesting that Tg was expressed not only in endothelial cells but also strongly in other cells including thymocytes (Figure (4(c))) and the germinal center (Figure (4(d))).

The expression of Tg was observed in only the sample from a 29-year-old patient (1/22, 4.5%). The average intensity of Tg in this sample was 0.1 (Table 1). Tg-positive Hassall's corpuscles were not found (Supplemental Table 1), while Tg staining was observed in thymocytes in this single sample (1/22, 4.5%; Supplemental Table 2 and Figure 4(c)).

**Figure 5:**
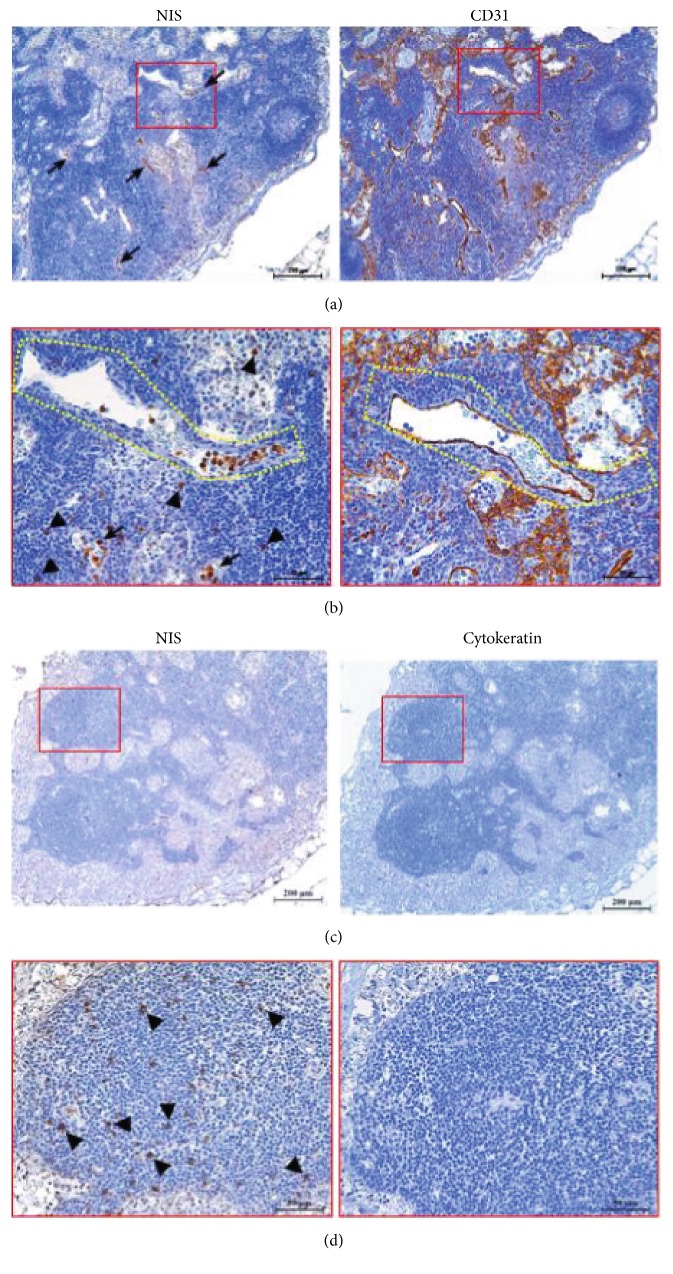
Expression of NIS protein in the Tg-positive thymus specimen. Sodium iodide symporter (NIS) is observed inside the vasculature stained with the CD31 antibody (a and b; arrows) and in thymocytes (b and d; arrowheads). Hassall's corpuscles were stained with cytokeratin. But Hassall's corpuscles were not observed in this thymus specimen. The low (a and c) and high (b and d) magnifications of the images are 100x and 400x, respectively. The scale bars represent 200 *μ*m (a and c) and 50 *μ*m (b and d).

NIS expression in the Tg-positive thymic tissue was unique (Figure 5). NIS-positive cells were observed to be independent of the cell shape inside vascular endothelial cells positive for CD31 in serial tissue sections (arrows in Figure 5(a) and (b)). Furthermore, NIS expression was also detected in thymocytes (arrowheads in Figure (5(b)) and (d)) located in thymic lobules. Unlike other NIS-positive cases, Hassall's corpuscles stained with the cytokeratin antibody or NIS antibody were not detected in the thymic tissue (Figure (5(c) and (d)). Furthermore, the expression of TSHR and TPO was not detected in the Tg-positive specimen.

### 3.3. Expression Incidence with Age

There were no significant differences in NIS, Tg, TSHR, or TPO expression among the age groups (Table 1). However, there was a trend toward increased frequency of TPO expression with increasing age. In the 20s, 30s, 40s, and over-50s age groups, TPO expression was observed in 0% (0/5), 16.7% (1/6), 20.0% (1/5), and 50.0% (3/6) of the samples (Table 1), respectively [odds ratio, 2.96; 95% confidence interval (CI), 0.883–9.903; *P* = 0.0786]. The intensity average was 0, 0.3, 0.4, and 1 with increasing age. NIS, TSHR, and Tg expression in thymocytes was not significantly different among the age groups (Supplemental Table 2).

### 3.4. mRNA Expression Levels of Thyroid-Related Genes

**Figure 6:**
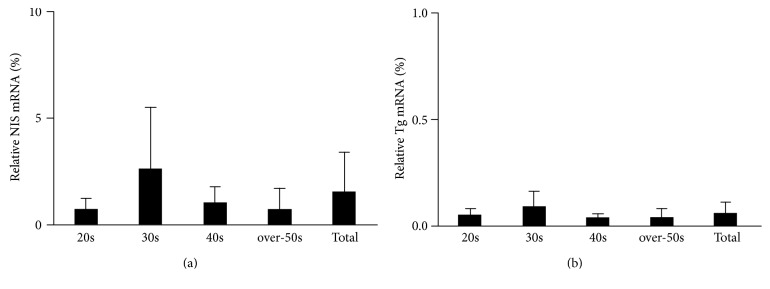
mRNA expression levels of thyroid-related genes in the thymus. The relative amount of mRNA in human thymic tissues is expressed as percentages of levels in normal thyroid tissues. The expression levels of both sodium iodide symporter (NIS) (a) and thyroglobulin (Tg) (b) mRNA are markedly lower in the thymus than in normal thyroid tissues. The mRNA levels were analyzed in the four age groups: 20s, 30s, 40s, and over-50s groups as percentages of each mRNA level found in normal thyroid tissues.

The levels of NIS and Tg mRNA expression were markedly reduced in thymic tissues compared with those in normal thyroid tissues (Figure 6). Among patients of all age groups (*n* = 13), NIS and Tg mRNA levels were 1.5% and 0.06%, respectively, compared with those in normal thyroid tissues. With regard to expression by age group, the percentages of NIS mRNA were 0.7%, 2.6%, 1.0%, and 0.7% in the 20s, 30s, 40s, and over-50s age groups, respectively. The percentages of Tg mRNA were 0.05%, 0.09%, 0.04%, and 0.04% in the 20s, 30s, 40s, and over-50s age groups, respectively. There was no significant difference in either NIS or Tg mRNA expression level among the age groups. We confirmed as a negative control that there was no signal with nuclease-free water instead of RNA template.

The mRNA expression level of Tg in the Tg-positive specimen was 0.01%, which was not higher than its expression level in other specimens. The mRNA expression level of NIS in the sample was 0.1%.

## 4. Discussion

In this study, expression of thyroid-related genes and proteins was detected in normal human thymic tissues. The expression of NIS and TSHR was most frequently detected in the thymus, whereas that of TPO was less frequently detected. The expression of Tg was observed in only one patient. To the best of our knowledge, only one report demonstrated the presence of thyroid-related epitopes, including NIS, TSHR, TPO, and Tg, in the thymus from two patients, where the expression of thyroid-related proteins was suggested to prime pre-T cells for generating self-tolerance against these epitopes [6].

NIS, TSHR, and TPO were distinctively localized to Hassall's corpuscles. This result is in agreement with the study of Vermiglio et al. who demonstrated using secondary ion mass spectrometry microscopy that iodine was located only in Hassall's bodies [8]. Hassall's corpuscles are concentrically arranged structures that differentiate from the medullary thymic epithelial cells after losing expression of the autoimmune regulator [9, 10]. An autoimmune regulator is a transcription factor that regulates the expression of numerous self-proteins in the medullary thymic epithelial cells and controls the intrathymic expression of autoantigens [4]. On the other hand, the presence of the variant TSHR transcript in RNA prepared from normal human thymus has been reported, suggesting induction of immunological tolerance [11]. Thus, the presence of these three proteins, NIS, TSHR, and TPO, in Hassall's corpuscles may represent vestiges of the T-cell repertoire. The function of thyroid-related proteins in the thymus after the completion of T-cell education remains unclear.

The expression of TSHR was frequently detected in the Hassall's corpuscles and thymocytes of normal thymic tissues. This result is in line with previous reports detecting the expression of TSHR in thymic epithelial cells and CD2^+^CD3^−^ and CD2^+^CD3^−^ thymocytes [12]. Murakami et al. [13] suggested that thymic hyperplasia is associated with Graves' disease and that thymic TSHR may act as an autoantigen involved in the pathophysiology of Graves' disease. Wortsman et al. [14] have reported that immunoglobulins from a patient with Graves' disease who had thymus enlargement bound to thymocytes and caused thymocyte proliferation. These results suggest the expression of TSHR in thymocytes and the effect by TSHR stimulation in thymocytes. In addition, our data on TPO expression in Hassall's corpuscles are consistent with those on TPO mRNA expression reported in the medullary thymic epithelial cells [12].

The expression of Tg was detected only in the thymus of a 29-year-old patient. IHC result showed the expression of Tg inside blood vessels. Serum Tg measurement with WBS has been widely used for postoperative follow-up of patients with DTC [15, 16]. We have clinically observed a few patients, particularly young patients, who showed elevated serum Tg levels without any evidence of recurrence and metastasis, suggesting Tg production elsewhere in the body. The normal thymus is a possible source of false-positive serum Tg. Tg mRNA levels, including that in the Tg-positive thymic sample, were low compared with levels in the normal thyroid tissue. The expression levels determined by qPCR may possibly be lower than those determined by IHC because qPCR utilizes whole cell extracts. In further study, it may get better results if in situ RT-PCR to define the location of the mRNA expression or qRT-PCR after laser-dissection for Hassall's corpuscles or cell fraction/sorting for thymocytes is performed. The mass of the thymus tissue we obtained was quite small to try to do cell fraction or sorting, because we obtained during central neck dissection adjunctive to total thyroidectomy.

We observed that the expression level of NIS protein was markedly lower in the thymus than in the normal thyroid. Similarly, the level of NIS mRNA expression measured using quantitative real-time PCR was quite lower in the thymus than in the normal thyroid. The ability of NIS to transport and concentrate iodide was reported to be decreased in extrathyroidal tissues [17]. This could explain the image findings of thymic uptake: the thymus was more frequently visualized on post-therapeutic WBS than on diagnostic WBS [18]. The thymus would suffer less damage, and some thymic tissues may remain compared with the thyroid after radioiodine therapy. This occurs because NIS expression is low in the thymus and extrathyroidal tissues. In addition, several iodine uptake-related factors in the thymus and extrathyroidal tissues are different from those regulated in the thyroid tissue [19].

Radioiodine uptake in the thymus has been reported in some young patients and can be misinterpreted as metastatic/recurrent thyroid cancer [4, 5]. Wilson et al. [4] reported the thymic uptake of radioiodine in 10/38 (26%) patients, of which nine were aged <50 years. There are several case reports on radioiodine uptake in the normal thymus without clinical or biological evidence of metastasis [4, 5, 20]. These reports imply the presence of NIS in the normal thymus.

In our study, IHC data showed that the expression of NIS was not increased in young patients compared with older patients. In addition, the levels of NIS gene expression were similar in all age groups. We found that thymus tissue structures tended to undergo involution in older patients. It has been reported that the thymic size decreases with increasing age in normal subjects [13]. The thymus starts shrinking and total fatty involution occurs around the age of 40 years, a time at which approximately 5% residual thymic tissue remains [19, 20]. Therefore, we suggest that the increased thymic uptake of radioiodine is not due to increased expression of NIS but rather due to more thymic tissue being present in young patients. This is the first report of a study analyzing the variation in the expression of NIS in the normal thymus according to age to find the evidence of the thymus uptake of radioiodine in young patients.

Several studies have reported ^18^F-FDG uptake in the thymus without any cancer metastasis or recurrence in the area [21–23]. This thymic ^18^F-FDG uptake mostly occurred in young patients and was described to be associated with larger glands. In addition, they found that the thymic ^18^F-FDG uptake related to hyperplasia and thymic condition and size changed after treatment or radioactive iodine ablation therapy [21, 23, 24].

In the future, it is necessary to correlate the levels of NIS expression in the thymus with the mediastinal uptake of radioiodine in WBS. These histological findings should be evaluated with clinical evidence. The patient with a histologically Tg-positive thymus should be further evaluated by measuring serum Tg to investigate the possibility that the thymus is a source of false-positive serum Tg. In this study, we could not obtain child thymus samples. The investigation in child samples below 10 years old and especially below 1 month would help to better understand whether the thyroid-related gene expression really evolves.

In conclusion, thyroid-related genes and proteins were expressed in human thymic tissues. The expression of NIS and TSHR was most frequently detected in the thymus, followed by TPO. The expression of Tg was found in only one patient. There were no significant differences in thyroid-related protein expression among the age groups. Therefore, we suggest that increased thymic uptake of radioiodine is not due to the increased expression of NIS but due to more thymic tissue being present in young patients.

## Supplementary Material

1) H&E staining in the supplementary materials and methods is related to supplementary figure 1. 2) Cell culture and 3) Western blotting in the supplementary materials and methods are related to the supplementary figure 2. The IHC method of supplementary table 1 and 2 is indicated in the materials and methods of original manuscript.

## Figures and Tables

**Table 1:** Expression of thyroid-related proteins in the thymus according to the ages.

	20s *n* = 5	30s *n* = 6	40s *n* = 5	Over-50s *n* = 6	Total *n* = 22
Positive sample number (%)					
NIS	4 (80%)	4 (66.7%)	4 (80%)	6 (100%)	18 (81.8%)
TSHR	4 (80%)	6 (100%)	3 (60%)	6 (100%)	19 (86.4%)
TPO^∗^	0 (0%)	1 (16.7%)	1 (20%)	3 (50%)	5 (22.7%)
Tg	1 (20%)	0 (0%)	0 (0%)	0 (0%)	1 (4.5%)

Average intensity					
NIS	1.8	1.8	1.8	2	1.9
TSHR	1.6	2.2	1.4	2.2	1.9
TPO	0	0.3	0.4	1	0.5
Tg	0.6	0	0	0	0.1

The expression of NIS, TSHR, TPO, and Tg was detected in the thymic tissues by immunohistochemistry (IHC). The positive IHC percentages and the average intensity were analyzed in the four age groups: 20s, 30s, 40s, and over-50s groups. The intensity of IHC staining was evaluated using a 4-point scoring system: 0, 1, 2, or 3 (for none, weak, moderate, or strong staining, resp.).

^∗^Odds ratio of positive TPO sample numbers, 2.96; 95% CI, 0.883–9.903; *p* = 0.0786.
