# Supplementary material for: Thyroid-Related Protein Expression in the Human Thymus
Source: Int J Endocrinol. 2017 Mar 2;2017:8159892. doi: 10.1155/2017/8159892 (PMC5353971; doi:10.1155/2017/8159892)

1. Supplemental Materials and Methods

1.1. H&E Staining. Surgical specimens of the thyroid and thymic tissues were paraffin-embedded for H&E staining and immunohistochemistry (IHC). Paraffin-embedded tissues sections (4 μm) were stained with hematoxylin (Polyscience, Inc. Warrington, PA) for 40 s and with eosin (Sigma-Aldrich, St Louis, MO) for 30 s. The tissue sections were examined under a light microscope (Olympus BX51; Olympus, Center Valley, PA) after mounting with Permount mounting medium (Fisher Scientific, Miami, FL).

2.1. Cell culture. Human papillary thyroid (TPC-1) cell lines were kindly provided from Dr. Shunichi Yamashita and Dr. Norisato Mitsutake (Nagasaki University Graduate School of Biomedical Sciences, Nagasaki, Japan). TPC-1 and FRO cells in RPMI 1640 medium (Welgene, Daegu, Korea) supplemented with non-heat-inactivated 5% fetal bovine serum (FBS; Invitrogen, Grand Island, NY) and 1× antibiotics (Invitrogen) were grown.

2.1. Western blotting. Grained thymus tissues or TPC-1 cells were lysated in radio-immunoprecipitation assay (RIPA) buffer (Sigma-Aldrich) containing protease inhibitor cocktail (Roche Diagnostics, Basel, Switzerland), and the insoluble pellets were removed by centrifugation at 8000 ×g for 10 min at 4°C. Proteins in the cell lysates were quantified using BCA protein assay kits (Thermo Scientific, Palm Springs, CA). Twelve microgram of proteins mixed with polyacrylamide gel electrophoresis sample buffer (Invitrogen) and NuPAGE reducing agent (Invitrogen) was separated in 8% SDS-PAGE mini gels and transferred to PVDF membranes (GE healthcare life sciences, Marlborough, MA). Membranes were blocked in 5% skim milk for 1 hr at room temperature, and incubated with following primary antibodies for NIS (goat polyclonal antibody (sc-48052)) for 1.5 hr at room temperature or rabbit anti-GAPDH (Abclon, Guro-gu, Seoul, South Korea). The membranes were incubated in Horseradish

peroxidase (HRP)-conjugated anti-rabbit secondary antibodies (Invitrogen). Bound secondary antibodies were detected by using ECL detection reagents (Roche, Nutley, NJ).

2. Supplemental Figure Legends

SUPPLEMENTAL FIGURE 1: Microscopic findings in human thymic tissues. Hematoxylin and eosin (H&E) staining was performed in the thymic tissues: representative 24-year-old (A), 39-year-old (B), 46-year-old (C) and 55-year-old patient (D) images are shown. All images are magnified 40x, and the scale bars represent 500 μm.

SUPPLEMENTAL FIGURE 2: NIS protein expression in the thymus tissue by Western blot. The expression of NIS protein was examined by Western blot analysis. The expression of NIS protein is shown in representative thymus samples in the four age groups (20s, 30s, 40s, and over 50s groups). TPC-1 cells were used as a positive control for NIS expression. Glycosylated (87 - 110 kDa) NIS proteins and non-glycosylated (approximately 50 kDa) NIS proteins are indicated with black arrow and white arrows, respectively. GAPDH was used as an internal control.

SUPPLEMENTAL TABLE 1: Expression of thyroid-related proteins in Hassall's corpuscles of the thymus according to the ages.

	20s<br>n = 5	30s<br>n = 6	40s<br>n = 5	Over 50s<br>n = 6	Total<br>n = 22
NIS	59%	66.7%	80%	88%	71.2%
TSHR	70%	97.5%	51.1%	100%	79.7%
TPO	0%	14.2%	14.6%	27.5%	14.1%
Tg	0%	0%	0%	0%	0%

The expression of NIS, TSHR, TPO, and Tg was detected in the thymic tissues by immunohistochemistry (IHC). The positive Hassall's corpuscles (%) were analyzed in the four age groups: 20s, 30s, 40s, and over 50s groups.

The positively stained and total Hassall's corpuscles were counted in the thymic tissues, and expressed as the percentage of positive Hassall's corpuscles.

SUPPLEMENTAL TABLE 2: Expression of thyroid-related proteins in thymocytes of the thymus according to the ages.

	20s<br>n = 5	30s<br>n = 6	40s<br>n = 5	Over 50s<br>n = 6	Total<br>n = 22
NIS	1<br>(20%)	2<br>(33.3%)	1<br>(20%)	1<br>(16.7%)	5<br>(22.7%)
TSHR	2<br>(40%)	5<br>(83.3%)	3<br>(60%)	5<br>(83.3%)	15<br>(68.2%)
TPO	0<br>(0%)	0<br>(0%)	0<br>(0%)	0<br>(0%)	0<br>(0%)
Tg	1<br>(20%)	0<br>(0%)	0<br>(0%)	0<br>(0%)	1<br>(4.5%)

The expression of NIS, TSHR, TPO, and Tg was detected in the thymic tissues by immunohistochemistry (IHC). The positive sample number and the positive percentages in thymocytes of the thymic samples were analyzed in the four age groups: 20s, 30s, 40s, and over 50s groups.

SUPPLEMENTAL FIGURE 1

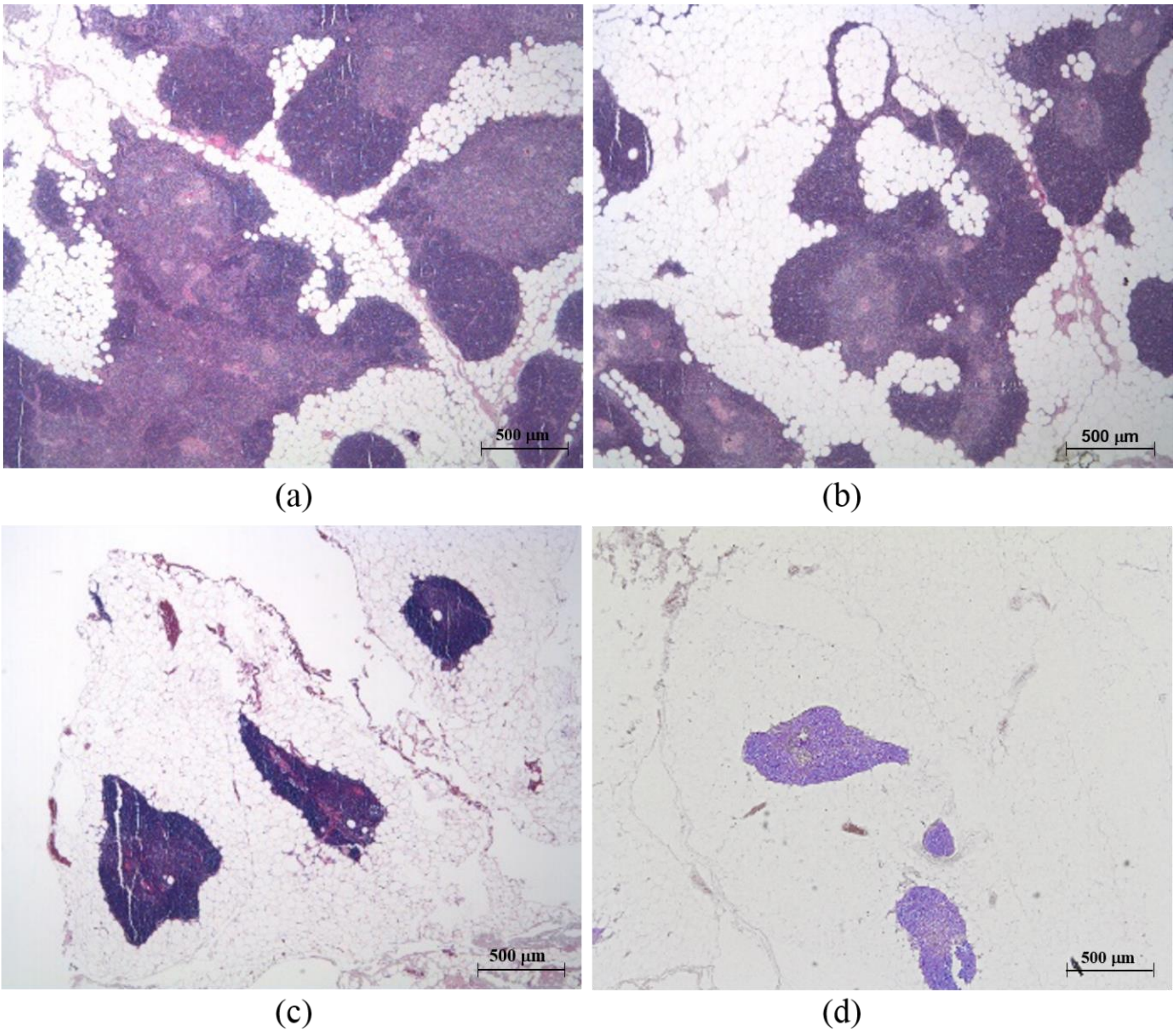

SUPPLEMENTAL FIGURE 2

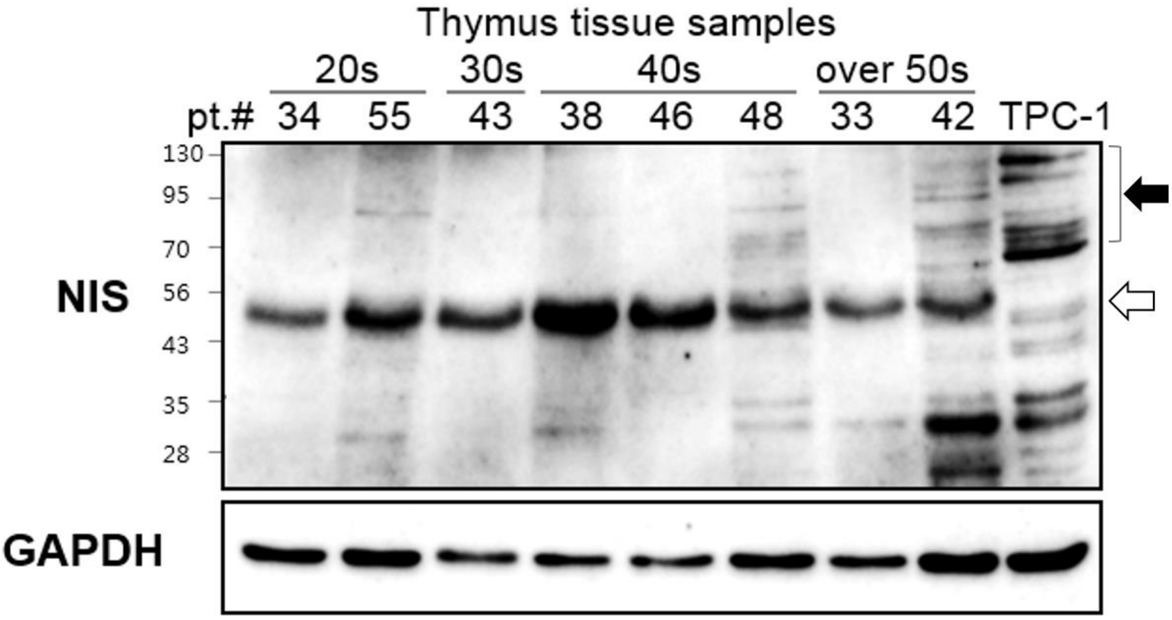

Supplement: Supplementary file 1 — 1) H&E staining in the supplementary materials and methods is related to supplementary figure 1. 2) Cell culture and 3) Western blotting in the supplementary materials and methods are related to the supplementary figure 2. The IHC method of supplementary table 1 and 2 is indicated in the materials and methods of original manuscript. [file 8159892.f1.pdf]
